# The impact of pedagogical beliefs on the adoption of generative AI in higher education: predictive model from UTAUT2

**DOI:** 10.3389/frai.2024.1497705

**Published:** 2024-10-17

**Authors:** Julio Cabero-Almenara, Antonio Palacios-Rodríguez, María Isabel Loaiza-Aguirre, Paola Salomé Andrade-Abarca

**Affiliations:** ^1^Department of Didactics and Educational Organisation, University of Seville, Seville, Spain; ^2^Department of Economics and Business Sciences, Private Technical University of Loja, Loja, Ecuador

**Keywords:** artificial intelligence, higher education, professor, educational technology, UTAUT2

## Abstract

Artificial Intelligence in Education (AIEd) offers advanced tools that can personalize learning experiences and enhance teachers’ research capabilities. This paper explores the beliefs of 425 university teachers regarding the integration of generative AI in educational settings, utilizing the UTAUT2 model to predict their acceptance and usage patterns through the Partial Least Squares (PLS) method. The findings indicate that performance expectations, effort expectancy, social influence, facilitating conditions, and hedonic motivation all positively impact the intention and behavior related to the use of AIEd. Notably, the study reveals that teachers with constructivist pedagogical beliefs are more inclined to adopt AIEd, underscoring the significance of considering teachers’ attitudes and motivations for the effective integration of technology in education. This research provides valuable insights into the factors influencing teachers’ decisions to embrace AIEd, thereby contributing to a deeper understanding of technology integration in educational contexts. Moreover, the study’s results emphasize the critical role of teachers’ pedagogical orientations in their acceptance and utilization of AI technologies. Constructivist educators, who emphasize student-centered learning and active engagement, are shown to be more receptive to incorporating AIEd tools compared to their transmissive counterparts, who focus on direct instruction and information dissemination. This distinction highlights the need for tailored professional development programs that address the specific beliefs and needs of different teaching philosophies. Furthermore, the study’s comprehensive approach, considering various dimensions of the UTAUT2 model, offers a robust framework for analyzing technology acceptance in education.

## 1 Introduction

Possessing a belief entail possessing a perceived truth, which subsequently dictates the subject’s actions and behavior in both advantageous and disadvantageous manners. The rapid pace of cultural, social, and technological transformation necessitates a reevaluation of personal beliefs, much like values, within the dynamic social reality we inhabit.

Beliefs, in their most expansive sense, wield transformative power over our cognition, opinions, competencies, and attitudes. They extend beyond mere personal constructs and are significantly influenced by the broader cultural milieu. As Jiménez (2019, p. 16) aptly articulates, “Beliefs are general ideas that we have about phenomena; they help us interpret, predict and control the events that occur and thus make decisions.” This conceptualization of beliefs is paramount in understanding their role in shaping our actions and behaviors, particularly for educators who must recognize that their pedagogical choices are profoundly influenced by their beliefs.

For a considerable duration (Brody and Day, 1993), it has been posited that in the realm of teaching, the significance and value that educators ascribe to their beliefs delineate the meaning of education and its various components (such as content, technology, didactic strategies, evaluation methods, etc.), and consequently influence their conduct in the educational process, their perception of it, and the nature of their interactions with students. Clark and Peterson (1986), pioneers in this research domain, assert that teachers’ cognitive frameworks impact their perceptions of students, their instructional planning, and their classroom behavior.

Montanares and Junod (2018, p. 94) further propose that “knowledge of teachers’ beliefs is important to the extent that it allows teachers a greater degree of awareness, responsibility and control in the choices of epistemological teaching models.”

The formation of teachers’ beliefs is influenced by a diverse array of variables, encompassing their professional experiences as educators and researchers, the nature of their training, their pedagogical education, ideological inclinations, interactions with colleagues, and the characteristics and ideology of the educational institution and community in which they operate (Arancibia-Herrera et al., 2024; Palacios-Rodríguez et al., 2023).

From the perspective of didactic research, investigations into beliefs have pursued various lines and orientations, including teaching strategies (Montanares and Junod, 2018; Cabero-Almenara et al., 2024), instructional practices (Rodríguez-Sosa and Solis-Manrique, 2017; Al-Adwan & Al-Debei, 2024), discipline-specific pedagogy (Casimiro, 2022), inclusive education (Friesen et al., 2023), the pedagogical utility of questioning (Mante-Estacioa and Tupas, 2023), STEM education (Alghamdi, 2023), and the role of emotions in professional teaching practices (Vettori et al., 2022).

A significant research trajectory has also examined beliefs about educational possibilities and the integration of Information and Communication Technologies (ICT). This research has evolved to focus on specific analyses, such as perceptions regarding the TPACK model’s dimensions in ICT integration (Ifinedo et al., 2020), the efficacy of video in language instruction (Waluyo and Apridayani, 2021), and attitudes toward incorporating ICT into educational practice (Li et al., 2019; Hoareau et al., 2021). Additionally, studies have explored how beliefs about technological competencies influence ICT integration (Cheng et al., 2022), the potential of ICT to support students with dyslexia (Bice and Tang, 2022), its effectiveness in lower education levels (Hoareau et al., 2021), and its applicability across various disciplines (García et al., 2022).

To summarize the significance of teachers’ beliefs concerning ICT application, Tondeur et al. (2017) conducted a meta-analysis and concluded: (1) a bidirectional relationship exists between pedagogical beliefs and ICT usage, (2) beliefs often act as perceived barriers, (3) specific beliefs are linked to specific types of ICT usage, (4) beliefs play a crucial role in professional development, and (5) the school context significantly influences beliefs about ICT.

Related to the theme of beliefs is the work on the “degree of acceptance of technologies” by teachers. Various models have been employed to analyze technology acceptance among potential users. The initial model, the Technology Acceptance Model (TAM) formulated by Davis (1989), posits that the intention to use technology is influenced by two primary dimensions: perceived usefulness and perceived ease of use, which in turn affect attitudes toward ICT, determining intentions to use and actual usage. This model has been utilized to analyze different technologies, such as virtual training (Rodríguez-Sabiote et al., 2023), augmented reality (Barroso et al., 2018), and immersive reality (Cabero et al., 2023; Palacios-Rodríguez et al., 2024a).

In contrast to the Technology Acceptance Model (TAM), Venkatesh et al. (2003) synthesized various proposed acceptance models, including TAM, to develop the Unified Theory of Acceptance and Use of Technology (UTAUT). This model aims to elucidate the acceptance and utilization of technology, predicated on four primary dimensions: performance expectancy, effort expectancy, social influence, and facilitating conditions. Subsequently, Venkatesh et al. (2012) refined the model, introducing the UTAUT2, which incorporates three additional dimensions: hedonic motivation (the pleasure derived from using the technology), price value, and the degree of automatic technology use. This reformulated model has garnered increasing adoption among researchers, as noted by Tamilmani et al. (2021) and García et al. (2022), compared to its predecessors.

**Figure 1:**
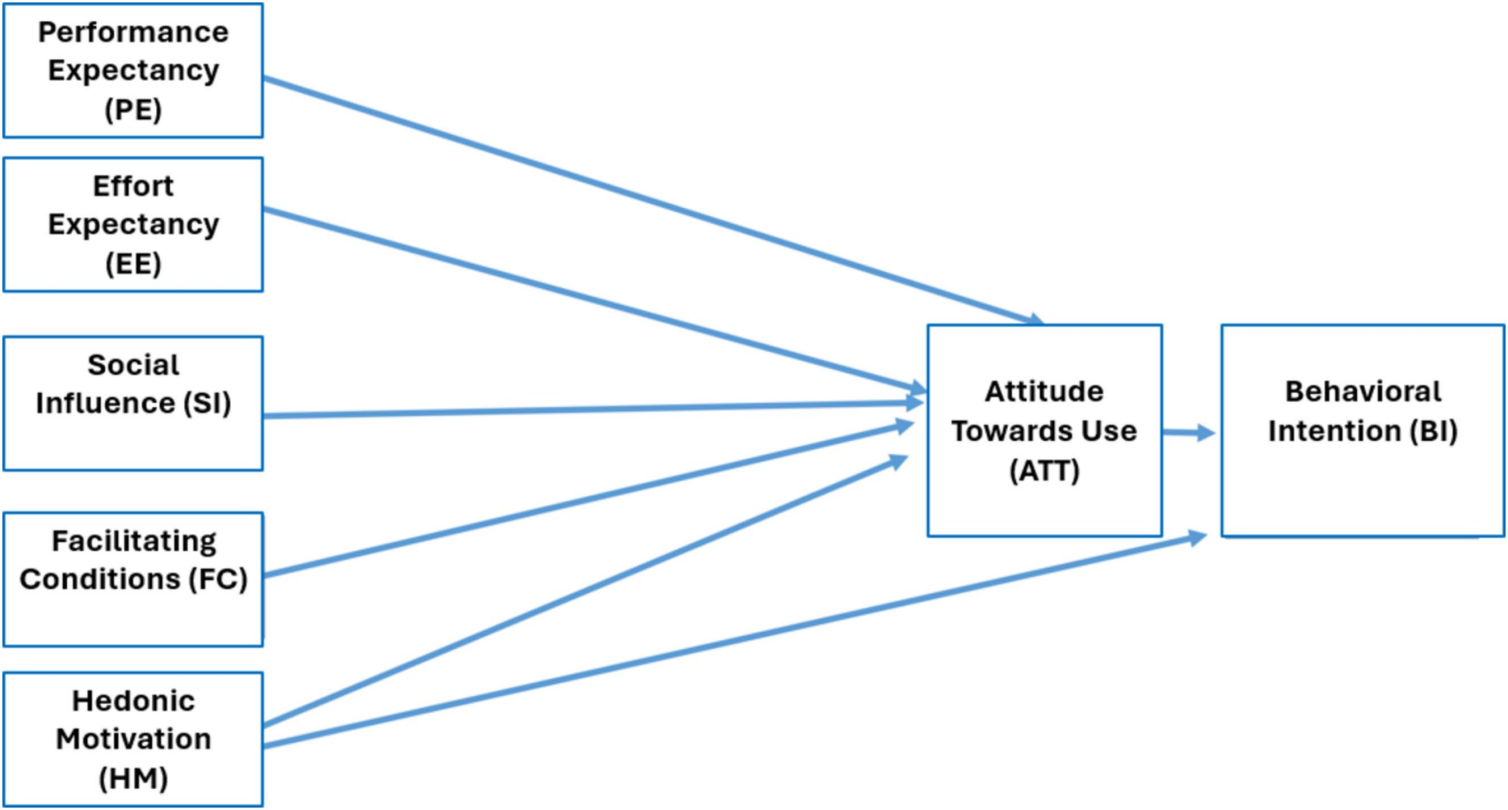
UTAUT2 model used in the study.

It is noteworthy that our study exclusively considers the first of the new variables introduced in UTAUT2—hedonic motivation. The variables of price value and automatic technology use were deemed irrelevant for the specific objectives of our research, thus configuring the model as depicted in Figure 1.

Understanding, according to various studies (Gansser and Reich, 2021; Al-Adwan and Al-Debei, 2024; Marikyan and Papagiannidis, 2023), the following dimensions are articulated within the UTAUT and UTAUT2 frameworks:

1. Performance Expectancy (PE): The degree to which an individual believes that the use of Artificial Intelligence in Education (AIEd) enhances their performance in relevant activities.
2. Effort Expectancy (EE): The degree to which an individual perceives that using AIEd will be free from excessive effort.
3. Social Influence (SI): The extent to which an individual perceives pressure from significant others (e.g., family, friends, colleagues) to adopt AIEd.
4. Facilitating Conditions (FC): The degree and availability of resources and support that facilitate the adoption and use of AIEd.
5. Hedonic Motivation (HM): The pleasure or enjoyment derived from using AIEd.
6. Attitude Toward Use (ATT): An individual’s intention to employ AIEd in their educational practices.
7. Behavioral Intention (BI): The extent to which an individual uses AIEd in their professional teaching activities.

It should be noted that the UTAUT and UTAUT2 models have been widely employed to assess the acceptance of various technologies. Within the UTAUT framework, research has explored the acceptance of the metaverse (Lee and Kim, 2022), mobile devices (Mojarro et al., 2019), and virtual reality (Ustun et al., 2023). Meanwhile, UTAUT2 has been applied to analyze the acceptance of technologies such as augmented reality (Huang, 2020), the metaverse (Al-Adwan and Al-Debei, 2024), virtual training platforms (Zacharis and Nikolopoulou, 2022), artificial intelligence (Gansser and Reich, 2021), and its educational applications (Strzelecki, 2023).

In short, these models enable us to comprehend whether teachers are inclined to use Educational Artificial Intelligence (AIEd), the extent of its usage, and the factors influencing these decisions, such as peer opinions, prior experiences, perceived value, and ease of use.

The recent advancements in Artificial Intelligence, particularly since the introduction of ChatGPT-3, have significantly impacted educational research. This progress has spurred investigations into attitudes, acceptance levels, necessary training, and the role of teachers’ beliefs in shaping students’ utilization of AI in educational settings. The importance of training educators in AI for both pedagogical and research purposes has also been highlighted (Alenezi et al., 2023; Tongfei, 2023; González-Mayorga et al., 2024; Temitayo et al., 2024).

Teacher beliefs are influenced by age, as demonstrated by Yuk and Lee (2023), who explored the experiences, perceptions, knowledge, concerns, and intentions of Generation Z students compared to older generations of educators. Their findings indicate generational differences in the adoption of technology in teaching.

Regarding AI, Adekunle et al. (2022) found that teachers’ confidence in teaching AI predicts their intention to incorporate AI into their instruction, suggesting that educators’ beliefs about AI’s usefulness and educational relevance are crucial. However, these beliefs are not uniform and vary depending on the discipline and educational level (Delgado et al., 2024).

From a psychoeducational perspective, two contrasting positions regarding conceptions of learning and teaching are prevalent: behaviorist and constructivist perspectives. The behaviorist approach suggests that knowledge is transmitted to the learner, while the constructivist perspective posits that knowledge is constructed subjectively and through social interaction (Arancibia, 2022). Choi et al. (2023) found that teachers with constructivist beliefs are more likely to integrate AI into their teaching than those with transmissive orientations.

Furthermore, studies have shown that teachers’ beliefs about learning impact their use of ICT in teaching and the frequency of its use. This includes research on general ICT usage (Prestridge, 2017; Bahçivan et al., 2018; Li et al., 2019; Arancibia-Herrera et al., 2024; Palacios-Rodríguez et al., 2024a, 2024b) and specific technologies such as mixed reality (Marín et al., 2023), the Moodle platform (Arancibia, 2022), and digital whiteboards (Burke et al., 2018).

## 2 Materials and methods

### 2.1 Research objectives

The research was carried out in the 2023–24 academic year and pursued three general objectives:

- Teachers must know the degree of acceptance of the AIEd.
- Determine if their constructivist and transmissive perspective on teaching determines the degree of acceptance.
- Validate the diagnostic instrument is used reliably.

**Figure 2:**
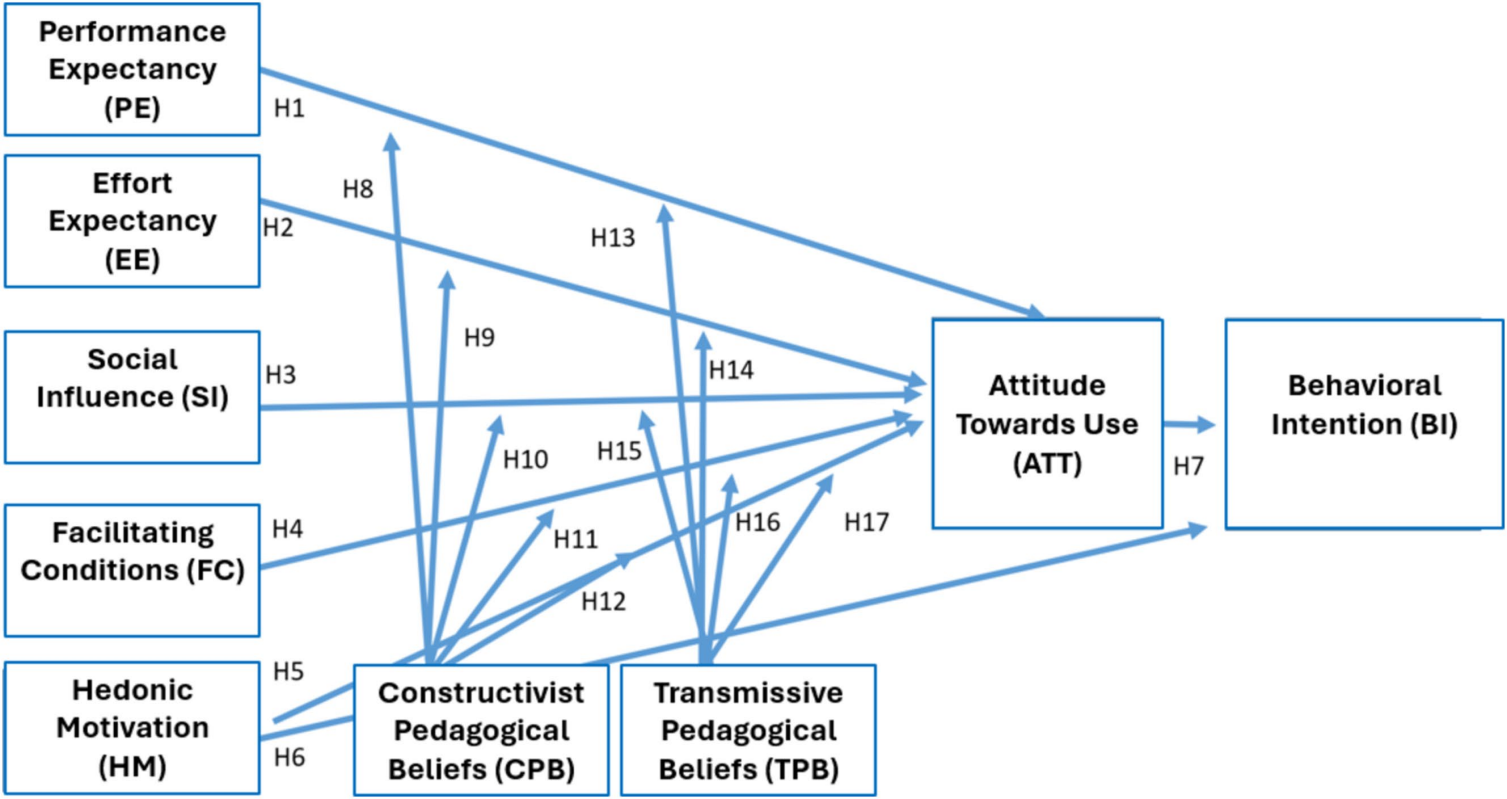
Model of the degree of acceptance of the AIEd and pedagogical beliefs of teachers.

To respond to these objectives, an “*ex post facto*” study was developed (Hernández-Sampieri and Mendoza, 2018), constructing the model presented below (Figure 2).

The model allowed the following hypotheses to be formulated:

1. HE1 Performance Expectancy (PE) positively predicts AIEd Behavioral Intention (BI).
2. HE2 Effort Expectancy (EE) positively predicts the AIEd Behavioral Intention (BI).
3. HE3 Social Influence (SI) positively predicts AIEd Behavioral Intention (BI).
4. HE4 Facilitating Conditions (FC) positively predict the AIEd Behavioral Intention (BI).
5. HE5 Hedonic Motivation (HM) positively predicts the intention to use AIEd.
6. HE6 Hedonic Motivation (HM) positively predicts AIEd Behavioral Intention (BI).
7. HE7 Attitude Toward Use (ATT) positively predict AIEd Behavioral Intention (BI).
8. HE8-H9-H10-H11-H12: Transmissive Pedagogical Beliefs (TPB) have a positive influence UTAUT2 dimensions.
9. HE13-H14-H15-H16-H17: Constructivist pedagogical beliefs have a positive influence UTAUT2 dimensions.

### 2.2 Sample

The research sample consisted of 425 professors from the Private Technical University of Loja (UTPL, Ecuador), of whom 233 (54.8%) were men and 192 (45.2%) were women, the rest, did not want to share the gender. The majority were between 31 and 40 years old (*f* = 163, 38.4%) and between 41 and 50 (*f* = 157, 36.9%). Professors who belonged to different areas of knowledge (Table 1).

**Table 1:** Area of knowledge of the teaching staff.

Areas of knowledge	*N*	%
Lost	6	1.4
Arts and humanities	78	18.4
Sciences	68	16.0
Health sciences	54	12.7
Social and legal sciences	154	36.2
Engineering and architecture	65	15.3
Total	425	100.0

The participants carried out their professional activity in the UTPL’s face-to-face modality (*f* = 110, 25.9%), the distance modality (*f* = 201, 47.3%), or both modalities (*f* = 114, 26.8%).

Faculty, what questions did they rate themselves, from 0 to 10, on the technical and didactic mastery that they considered they had regarding ICT? They reached an average score of 8.04 regarding technical mastery, with a standard deviation of 1.41, and a mean of 8.04 concerning the didactic domain, in this case with a standard deviation of 1.48.

### 2.3 Instrument

The instrument was made up of three large blocks: the first collected information on the characteristics of the person who completed it: gender, age, faculty/center where they taught, perception of their technical mastery for the management of the ICT, and perception of its didactic domain for the incorporation of ICT in training; the second, which analyzed the degree of acceptance of the AIEd, created from the instruments developed by different authors (Huang, 2020; Al-Adwan and Al-Debei, 2024; Strzelecki, 2023); and the third, which sought to know the teacher’s transmissive or constructivist pedagogical belief, adopted from the work of Choi et al. (2023).

The instrument had 30 items in total: 5 for the first part, 25 for the second, and 10 for the third. It was also administered via the Internet.

## 3 Results

### 3.1 Degree of acceptance and pedagogical beliefs

The means and standard deviations obtained for all the questionnaire items will be presented initially (Table 2).

**Table 2:** Mean scores and standard deviations.

ITEMS	*M*	SD
PE1: I find that AIEd is helpful for learning	6.04	1.129
PE2: Using AIEd for learning increases productivity	5.93	1.271
PE3: Using AIEd for learning improves effectiveness	5.79	1.344
PE4: Using AIEd for learning improves academic performance	5.49	1.475
EE1: My interaction with the AIEd is clear and understandable	5.48	1.272
EE2: It would be easy for me to use AIEd proficiently.	5.97	1.112
EE3: I think it would be easy for me to understand how the AIEd programs are used.	6.05	1.013
EE4: Learning to trade with AIEd would be easy for me	6.00	1.000
SIl: People who influence my behaviour think I should use AIEd to develop learning.	4.98	1.759
SI2: People who are important to me think I should use AIEd to develop learning.	5.05	1.768
SI3: In general, university authorities have supported the use of AIEd for the development of learning.	5.55	1.398
SI4: In general, I am very supportive of using AIEd for learning.	5.62	1.499
FC1: I have the necessary resources to use the AIEd.	5.52	1.409
FC2: I have the necessary knowledge to use the AIEd.	5.28	1.360
FC3: AIEd is not compatible with other learning systems that I use	4.20	2.055
FC4: A particular person (or group) can help me with AIEd difficulties.	4.42	1953
HM: 1 Seeing the information in the AIEd is nice.	5.69	1.354
HM2: Seeing the information in the AIEd is entertaining.	5.68	1.400
HM3: Seeing the information in the AIEd is fun.	5.57	1.434
ATT1: Once you start using AIEd in learning, I find it difficult to stop using it.	5.08	1.637
ATT2: The study is more interesting in using AIEd in learning.	5.32	1.526
ATT3: Learning content using AIEd is a pleasant activity	5.37	1.505
BI1: I intend to continue using AIEd in future learning.	5.79	1.393
BI2: I will insist on using AIEd in my courses.	5.51	1.506
BI3: I plan to use the AIEd system in the future.	5.95	1.299
CPB1: Learning means students have ample opportunities to explore, discuss and express their ideas.	6.48	0.934
CPB2: Each student is unique or special and deserves an education adapted to their needs.	6.41	0.965
CPB3: The teacher needs to understand the student’s feelings.	6.32	1.073
CPB4: Good teachers always encourage students to think for themselves for answers.	6.62	0.777
CPB5: In good classrooms, there is a democratic and free environment that encourages students to think and interact.	6.61	0.763
TPB1: Keeping students confined to textbooks and desks is essential during the lesson.	3.97	2.208
TPB2: Learning to teach means practicing teachers’ ideas without questioning them.	3.04	2.358
TPB3: Teaching is simply telling, presenting or explaining the topic.	2.96	2.299
TPB4: Good teaching occurs when there are mainly explanations from teachers in the classroom.	4.64	2.035
TPB5: Teaching is about providing students with accurate and complete knowledge rather than encouraging them to discover it.	3.54	2.354

As can be seen, the average values achieved are generally above the average value of the scale, which was 3.5. At the same time, it can be noted that the scores achieved in the standard deviations, which in some cases exceed two points, reflect a substantial dispersion of the data and, therefore, of the answers offered by the teachers.

Table 3 presents the dimensions of the questionnaire section that analyzed the degree of acceptance of the technology, as well as the mean scores and standard deviation found.

**Table 3:** Means and standard deviations of the dimensions of the degree of acceptance.

Dimensions	M	SD
Performance expectancy (PE)	5.81	1.188
Effort expectancy (EE)	5.87	0.951
Social influence (SI)	5.30	1.328
Facilitating conditions (FC)	4.85	1.294
Hedonic motivation (HM)	5.64	1.329
Attitude toward use (ATT)	5.25	1.424
Behavioral intention (BI)	5.75	1.313

It should be noted that in all cases, the average scores exceed the value of 3.5, which suggests a high degree of acceptance of the AIEd by teachers and a high intention to use it (5.75).

Table 4 offers the average scores achieved in the dimensions referring to the analysis of the teachers’ constructivist and transmissive pedagogical beliefs. This clearly shows that teachers tend toward a constructivist style. As can be seen, there is mostly a perception that teachers assume constructivist positions for developing formative actions (6.49) versus transmissive actions (3.63). However, the high score achieved in the standard deviation found in the transmissive option should also be highlighted, which implies a high dispersion in the answers offered.

**Table 4:** Means and standard deviations of the dimensions referring to teachers’ pedagogical beliefs.

Dimensions	*M*	SD
Constructivist pedagogical beliefs (CPB)	6.49	0.733
Transmissive pedagogical beliefs (TPB)	3.63	1.972

### 3.2 Model validation

Structural analysis models have garnered increasing attention in social research due to their ability to explore both explicit and implicit variables, facilitating their integration in comprehensive models (Alaminos et al., 2015). These models provide a robust framework for examining complex relationships between multiple variables, making them especially useful in fields that require the analysis of latent constructs.

In the context of Structural Equation Modeling (SEM), two primary methodologies are commonly employed: covariance-based SEM and Partial Least Squares (PLS). The selection of methodology depends on the characteristics of the data and the research objectives. In this study, the PLS approach was chosen due to its flexibility, particularly its lack of reliance on the assumption of multivariate normality of the data, which is a prerequisite in covariance-based SEM methods. PLS is particularly suitable when dealing with small sample sizes and models with higher complexity, as it allows the estimation of parameters even when normality is not met (Hair et al., 2017).

For the implementation of the PLS approach, the SmartPLS software was used. This software is widely recognized in the field of SEM for its efficiency in handling PLS-based analysis. The analysis followed the standard phases typically associated with structural equation modeling using PLS, as described in the literature (Sampeiro, 2019). These phases include the specification of the measurement and structural models, the evaluation of the outer model (convergent and discriminant validity), and the assessment of the inner model (path coefficients and the explained variance).

The Cronbach’s Alpha coefficient was initially applied to evaluate the reliability of the different constructs contemplated in the proposed model (Table 5).

**Table 5:** Reliability according to Cronbach’s alpha.

	Cronbach’s alpha
Attitude Toward Use (ATT)	0.931
Behavioral Intention (BI)	0.937
Effort Expectancy (EE)	0.920
Facilitating Conditions (FC)	0.729
Hedonic Motivation (HM)	0.936
Performance Expectancy (PE)	0.937
Social Influence (SI)	0.835
Transmissive Pedagogical Beliefs (TPB)	0.935
Constructivist Pedagogical Beliefs (CPB)	0.871

According to several authors (Mateo, 2004), exceeding 0.7 indicates that all the levels obtained are adequate.

Regarding the loadings or simple correlations of the indicators with their respective constructs, the different values obtained are presented in Table 6. It is essential to remember that for an indicator to be considered part of a construct, it must have a loading greater than 0.7 (Carmines and Zeller, 1979).

**Table 6:** Loadings or simple correlations of the indicators with their respective construct.

	ATT	BI	CPB	EE	FC	HM	PE	SI	TPB
ATT1	0.903								
ATT2	0.964								
ATT3	0.945								
BI1		0.961							
BI2		0.928							
BI3		0.937							
CPB1			0.753						
CPB2			0.846						
CPB3			0.808						
CPB4			0.846						
CPB5			0.801						
EE1				0.790					
EE2				0.938					
EE3				0.943					
EE4				0.920					
FC1					0.817				
FC2					0.807				
FC3					0.701				
FC4					0.806				
HM1						0.892			
HM2						0.971			
HM3						0.959			
PE1							0.882		
PE2							0.936		
PE3							0.937		
PE4							0.913		
SI1								0.845	
SI 2								0.831	
SI 3								0.787	
SI4								0.804	
TPB1									0.869
TPB2									0.934
TPB3									0.904
TPB4									0.840
TPB5									0.901

As can be seen, all values have a loading greater than 0.7. Therefore, no items were eliminated during this phase of the analysis.

The next step involves analyzing the composite reliability (CR) related to the internal consistency of the indicators that examine the latent variables. This value allows us to determine if each indicator measures the same thing and whether the latent variable is well represented. The minimum appropriate value is considered to be 0.7. In addition, convergent validity is calculated to determine whether a group of indicators represents a single underlying construct. This value is calculated using the Average Variance Extracted (AVE). To ensure a good fit of the model, it is considered that the value of AVE must be greater than 0.5, which means that more than 50% of the variance of the construct is explained by the indicators (Bagozzi and Yi, 1988). The results are shown in Table 7.

**Table 7:** Composite reliability and average extracted variance of the model.

	CR	AVE
ATTITUDE Toward Use (ATT)	0.938	0.880
Behavioral Intention (BI)	0.937	0.888
Effort Expectancy (EE)	0.930	0.810
Facilitating Conditions (FC)	0.780	0.555
Hedonic Motivation (HM)	0.945	0.886
Performance Expectancy (PE)	0.938	0.841
Social Influence (SI)	0.847	0.667
Transmissive Pedagogical Beliefs (TPB)	0.947	0.793
Constructivist Pedagogical Beliefs (CPB)	0.880	0.658

Continuing with obtaining discriminant validity, which determines whether each established construct is significantly different from the others, two approaches are used: the Fornell-Larcker criterion (Table 8) and cross-factor loadings (Table 9).

**Table 8:** Fornell-Larcker criterion.

	ATT	BI	CPB	EE	FC	HM	PE	SI	TPB
ATT	0.938								
BI	0.848	0.942							
CPB	0.365	0.404	0.811						
EE	0.408	0.419	0.316	0.900					
FC	0.546	0.552	0.258	0.616	0.745				
HM	0.778	0.773	0.384	0.413	0.612	0.942			
PE	0.804	0.815	0.417	0.443	0.476	0.658	0.917		
SI	0.791	0.766	0.347	0.476	0.656	0.678	0.757	0.817	
TPB	0.270	0.129	−0.061	0.127	0.391	0.257	0.124	0.293	0.890

**Table 9:** Standardized regression coefficients (path coefficients).

	ATT	BI	CPB	EE	FC	HM	PE	SI	TPB
ATT		0.626							
BI									
CPB				0.325	0.281	0.401	0.426	0.366	
EE	−0.028								
FC	−0.031								
HM	0.363	0.286							
PE	0.362								
SI	0.305								
TPB				0.146	0.411	0.281	0.150	0.316	

The Fornell-Larcker criterion is based on the fact that the average extracted variance of a construct must be greater than the variance that said construct shares with the other constructs in the model. Similarly, the correlations between the constructs must be less (in absolute value) than the square root of the average variance extracted. This condition is verified by analyzing that the values on the diagonal, which correspond to the square root of the average extracted variance, are more significant than the values off the diagonal, which represent the correlations between constructs. This condition is met in all observed cases.

**Figure 3:**
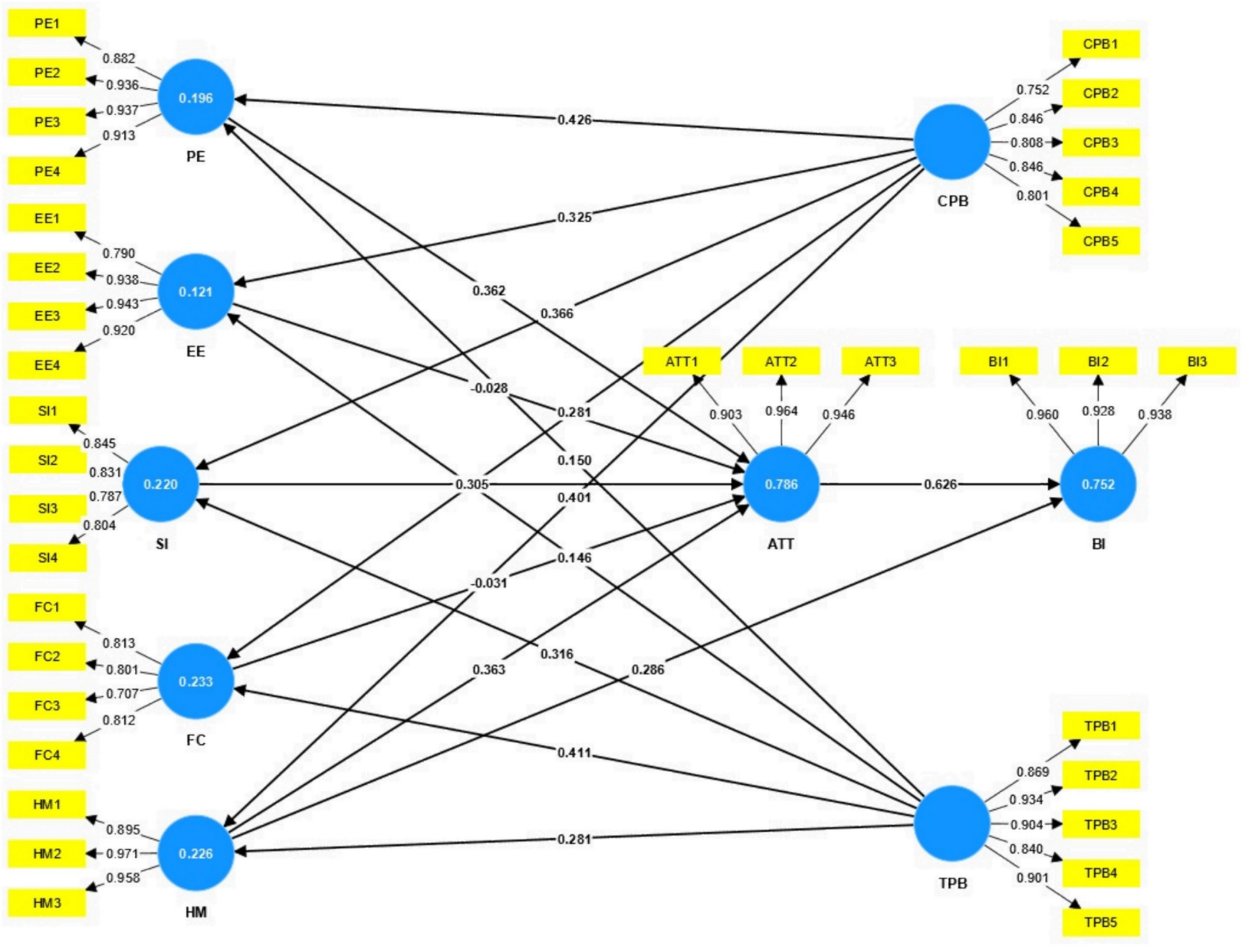
PLS model.

The analyses carried out up to this point lead to the conclusion that the items included in the questionnaire exhibit acceptable levels of reliability and show high consistency concerning the dimensions in which they are located in the model. Next, the formulated structural model is analyzed by generating standardized regression coefficients (path coefficients), Student’s t values and R2 (R-squared). These data provide information on the percentage of variance of the constructs explained by their predictor variables and, therefore, allow the viability of the developed model to be evaluated (Figure 3).

The data from the predictive model indicate that Behavioral Intention (BI) is 75.2%, explained by the rest of the variables, suggesting that these variables strongly influence use behavior. Similarly, Attitude Toward Use (ATT) is explained by 78.6% of the rest of the variables, indicating a significant influence of the variables on intention to use. Furthermore, it is observed that the constructivist and transmissive pedagogical belief variables positively impact the model.

The Student t-test is applied to the path values (path coefficients) using the Bootstrap technique to determine the significance of the scores achieved. This resampling method allows us to estimate the sample distribution and calculate confidence intervals for the path coefficients, which helps to evaluate their statistical significance more robustly (Table 8). All coefficients show significance *p* < 0.005, which implies statistically significant relationships between the analyzed variables.

Finally, to evaluate the structural model’s fit quality, we use the Standardized Root Mean Square Residual (SRMR) indicator, which registers a value of 0.061. This value is less than 0.08 (Bagozzi and Yi, 1988), which suggests a satisfactory fit of the model. Our study also reveals more significant loading in the model dimensions for teachers with constructivist pedagogical beliefs than those with transmissive beliefs regarding performance expectations, effort expectations, social influence, and facilitating conditions.

## 4 Discussion and conclusions

The conclusions of our study point in different directions. Firstly, the instrument’s reliability has been demonstrated to exhibit reasonably high levels when analyzing the dimensions identified from the UTAUT2 model and the constructivist and transmissive pedagogical perspectives. These values align with those found by Choi et al. (2023) and Cabero-Almenara et al. (2024).

Simultaneously, the model formulated to analyze the degree of teachers’ acceptance of AIEd proved reliable and valid. The results confirmed the significance of 16 of the 17 formulated hypotheses, with the exception of the hypothesis relating to Effort Expectancy (EE) in Attitude Toward Use (ATT). This discrepancy could be attributed to the ease of using generative AI tools and the lack of knowledge or training among teachers. Future research should consider directly addressing questions about the degree of technical and didactic mastery specifically related to AIEd, rather than to technologies in general.

It is essential to highlight that Attitude Toward Use (ATT) is the most significant and influential dimension concerning Behavioral Intention (BI). Therefore, the intention to use fundamentally determines and directs the actual use by the teacher. Moreover, the results indicate that teachers with constructivist beliefs are more likely to integrate AIEd into teaching than those with transmissive orientations. This finding corroborates the results achieved by Choi et al. (2023). However, in this study, the acceptance model used was the UTAUT2. Only in the case of Effort Expectancy (EE) did teachers with transmissive beliefs establish a higher score load, potentially indicating their perception of greater difficulty in using AIEd.

The findings of this study have both practical and theoretical implications for understanding AIEd. To our knowledge, this is one of the first empirical studies to address teachers’ perceptions of AIEd through the UTAUT2 technology acceptance model. Previous studies have utilized the TAM model of technology acceptance (Choi et al., 2023) or the UTAUT2 model with university students (Strzelecki, 2023).

This study developed a conceptual model derived from the UTAUT2 and integrated the potential significance of teachers’ transmissive or constructivist pedagogical beliefs for the use of AIEd. Through a structural equation modeling (SEM) analysis, it is posited that the proposed model offers an adequate explanation of teachers’ intentions to use AIEd. This study has highlighted the importance of considering Performance Expectancy (PE), Effort Expectancy (EE), Social Influence (SI), Facilitating Conditions (FC), and Hedonic Motivation (HM) in predicting the acceptance of AIEd and its impact on attitudes and behavioral intentions to use (Al-Adwan & Al-Debei, 2024).

The findings reveal that teachers with a more constructivist approach positively correlate with the perceived usefulness and effectiveness of AIEd, leading to a higher likelihood of adoption. Consequently, educational institutions must promote a constructivist-oriented pedagogical perspective while providing teacher training programs to familiarize them with AIEd and enhance their digital competencies for integration into the educational environment.

However, this research has limitations that should be addressed in future studies. Firstly, the extent to which respondents were exposed to AIEd was not considered. Additionally, the demographic characteristics of the teachers, such as age, gender, and subjects taught, were not included in the analyses but will be addressed in future work currently in preparation.

Finally, incorporating qualitative data would provide a more comprehensive investigation into the determinants influencing teachers’ acceptance of AIEd. Combining qualitative methods, such as focus group interviews or nominal group techniques, could enable researchers to unravel the underlying mechanisms involved in this acceptance process.

## Data Availability

The raw data supporting the conclusions of this article will be made available by the authors, without undue reservation.
